# Do adolescent girls’ education and friendships have independent effects on early pregnancy? Results of a mediation analysis from a longitudinal cohort study in Nairobi, Kenya

**DOI:** 10.1016/j.ssmph.2024.101618

**Published:** 2024-02-01

**Authors:** Christina Misunas, Erica Soler-Hampejsek, Beth Kangwana, Nicole A. Haberland

**Affiliations:** a University of California, Berkeley, USA; b Independent Consultant, Barcelona, Spain; c Population Council, Nairobi, Kenya; d Population Council, New York, NY, USA

**Keywords:** Adolescent girls, Adolescent pregnancy, Education, Literacy, Numeracy, School attendance, Friendships

## Abstract

**Background:**

Few studies have examined whether the effect of education on pregnancy and childbearing is due to the academic skills acquired or the social environment that schooling provides. This paper explores whether adolescent girls’ learning skills, school enrollment, grade attainment, and friendships affect risk of pregnancy, and whether friendships mediate the relationship between education and pregnancy.

**Methods:**

We draw on three waves of longitudinal data on adolescent girls aged 11–15 in Kibera, an informal settlement in Nairobi, Kenya between years 2015–2019. We use fixed effects regression models to estimate effects of girls’ learning skills, school attendance, grade attainment, and friendships on their probability of experiencing a pregnancy. We conduct mediation analyses to assess whether friendships mediate the relationship between education and pregnancy.

**Results:**

By round one (2015), 0.1 % of girls reported having experienced a pregnancy; by round three (2019), 6.3 %. Even after adjusting for friendships, we find that attending school decreases probability of pregnancy by nine percentage points; an additional year of schooling decreases probability of pregnancy by three percentage points; and a one standard deviation increase in numeracy decreases probability of pregnancy by one percentage point. Having any male friends who do not attend school increases girls' probability of experiencing a pregnancy by four percentage points; this association remains after adjusting for girls' education. However, out-of-school girls are far more likely to report out-of-school male friends. We find no evidence that other types of friendships affect girls’ probability of becoming pregnant.

**Conclusion:**

We find significant protective effects of school attendance, higher grade attainment and numeracy skills on girls' pregnancy, and that having close friendships with out-of-school males increases girls’ probability of pregnancy. We did not find evidence of meaningful mediation, suggesting that the protective effects of school attendance and learning remain regardless of any risk they may face from their friendships.

## Highlights

• Attending school, an additional year of schooling, and higher levels of numeracy decrease girls' probability of pregnancy.
• Having out-of-school male friends increases girls' probability of pregnancy.
• Other types of friendships do not affect girls' probability of becoming pregnant.
• Friendships do not mediate the relationship between education and pregnancy.

## 1 Introduction

While adolescent fertility rates have been on a downward trend, they remain high in many parts of the world (Sully et al., 2020). In low- and middle-income countries, an estimated 12 million girls aged 15–19 and 777,000 girls under age 15 give birth each year (Sully et al., 2020). The occurrence of pregnancy, childbirth, and motherhood during adolescence has been linked to a range of adverse schooling and health outcomes in the short- and long-term for girls and their children (Fall et al., 2015).

### 1.1 Influence of girls' education on the risk of pregnancy

Research on factors influencing the risk of pregnancy among adolescent girls has consistently demonstrated an inverse relationship between girls’ education and their age at first birth (Black, Devereux, and Salvanes 2008; Breierova & Duflo, 2004; Bledsoe et al., 1999; National Research Council (US) Working Group on the Social Dynamics of Adolescent Fertility 1993). Yet despite extensive research on this topic, most studies have only demonstrated associations rather than causality, largely due to the bidirectional nature of the relationship. For example, a 2019 systematic review identified 22 studies that looked at the causal effects of education (grade attainment) on age at first birth (Psaki et al., 2019). The authors find limited causal evidence across these studies that increased schooling delays age at first sex and mixed effects on delaying age at first birth (Psaki et al., 2019).

Furthermore, few studies have explored how other important aspects of girls' education beyond grade attainment, such as academic skills such as literacy or numeracy, may be causally related to pregnancy (Marteleto et al., 2008; Psaki et al., 2019). Measuring the causal effects of learning skills or academic performance might provide nuance on the links between girls’ education and sexual and reproductive health outcomes, particularly in settings where school access and attendance are high or near universal or where there may be high rates of grade repetition (Marteleto et al., 2008).

Moreover, while some studies have found that school enrollment, attendance, and performance provide a protective effect, very little empirical evidence exists on the mechanisms through which girls' education impacts their risk of pregnancy (Psaki et al., 2019; Zahra et al., 2022). Exploring and identifying these mechanisms is essential for understanding and more accurately projecting the impacts of and return on investments in girls’ education (Psaki et al., 2019; Zahra et al., 2022).

There are many individual and contextual pathways through which education may theoretically affect girls' risk of pregnancy and childbirth (Basu, 2002). For example, the classroom might provide a structured environment and the time commitment of attending school may limit girls' exposure to wanted or unwanted sexual activity (Bandiera et al., 2018). In settings where marriage typically precedes childbearing, schooling may provide an alternative to early marriage and thus delay girls’ age at first sex and first birth (Marphatia et al., 2020).

In attending school and interacting with formal institutions, students might also gain skills that help them successfully navigate health systems and access services, including those related to their sexual and reproductive health. Learning, specifically the development of literacy skills, may improve girls' ability to understand sexual and reproductive health messages. Other potential pathways through which schooling and learning outcomes might influence girls' risk of pregnancy are through the social environment that schooling provides and the connections that it fosters to people, particularly peers, who might influence girls’ attitudes and behaviors.

### 1.2 Influence of girls' education on their social networks

Schooling can affect girls' risk of pregnancy through the influence of social norms and peer behavior as well as by helping to determine which peers are influential. Girls' school attendance and academic performance have the potential to shape the composition of their social networks in important ways. At a basic level, the classroom might serve as a social space and provide a setting for girls to befriend other students who are likely to be of similar age, including boys and young men. Likewise, the school environment might broaden girls' social networks if in-school friends facilitate introductions to individuals outside the classroom or the school. It is also possible that since school attendance entails physical presence in the classroom, schooling can also limit students’ exposure to and interactions with out-of-school peers and older adolescents or adults.

In addition to school attendance, girls' academic performance might also serve as a sorting mechanism for their friendships and their social networks more broadly. Some evidence has shown that adolescents are likely to maintain friendships with students who are academically similar and that self-sorting on the basis of academic performance is common, particularly among secondary school students (Flashman, 2012; Lucas, 1999). Those friendships might directly influence girls' risk of pregnancy and, in turn, might mediate any effects of girls’ education.

### 1.3 Influence of girls' social networks on the risk of pregnancy

A growing number of studies have demonstrated the nontrivial effects of adolescent girls’ social networks on their sexual and reproductive health (Barker et al., 2019; Fletcher, 2007; Fletcher & Yakusheva, 2016; Mrug, Borch, and Cillessen 2011; Shakya et al., 2020; Sieving et al., 2006). The social spillover effects of peers relative to other contextual influences have also been explored: in a recent US-based study, Barker et al. also found that, relative to schools and to neighborhoods, peer groups accounted for more of the total variation in age at sexual initiation among adolescents (Barker et al., 2019).

Girls’ social networks may influence their risk of pregnancy in many ways, some of which are unique to the adolescent stage of development. During the transition to adulthood, adolescents experience changes in their broader social networks and an increase in their interactions with peers (Brown et al., 1997); in some cases, the nature and dynamics of their social relationships evolve from platonic to romantic (Thornton, 1990). At a basic level, simply having more friends of another sex during adolescence has been shown to be associated with earlier sexual initiation (Billari & Mencarini, 2004).

Evidence from developmental neuroscience also indicates that during adolescence, the brain is highly plastic and undergoes a major “social reorientation,” making adolescents particularly sensitive to norms and influences from peers (Telzer et al., 2018).

In terms of the impact of the normative environment, research has shown that adolescents' perceptions of norms among their peers can influence their own health views and behaviors (Evans et al., 1995), including around sexual initiation, contraceptive use, and pregnancy (Fletcher & Yakusheva, 2016; Mrug et al., 2011). If one's friends engage in sex, become pregnant, or get married, these behaviors might become more normative, increasing one's sexual risk. Indeed, among adolescents in the United States, those with a higher proportion of friends who were sexually experienced had higher odds of sexual initiation relative to their peers (Sieving et al., 2006). A study in Honduras found that early childbearing in a girl's social network increased her likelihood of adolescent childbearing (Shakya et al., 2020). Inequitable gender norms among adolescents may also condone males' entitlement to sex, or impede females' ability to insist on condom use (Okumu et al., 2023; Pulerwitz & Barker, 2008; Shai et al., 2012).

Another potential mechanism through which social networks might influence risk of pregnancy is through the sharing of knowledge based on information and lived experiences (Fletcher & Yakusheva, 2016). For example, having friends who share information and reinforce messaging around continuing education, delaying sexual initiation and family formation, and contraceptive use may foster these behaviors. Ali and colleagues find that among students in the United States, a 10 % increase in the proportion of classmates who use contraception increases the likelihood of individual contraception use by 5 % (Ali, Amialchuk, and Dwyer 2011). Another U.S.-based study found that having friends who have attitudes not accepting of early pregnancy reduced the likelihood of subsequent adolescent pregnancy, and having friends with negative attitudes about contraception decreased likelihood of subsequent contraceptive use (Dehingia, Barker, and Raj 2022).

### 1.4 Country context

In 2022, the age specific fertility rate in Kenya was 73 live births per 1000 women aged 15 to 19; in Nairobi, 57 live births per 1000 women aged 15 to 19 (Kenya National Bureau of Statistics and The DHS Program ICF, 2023). This represents a decline from year 2014–96 live births per 1000 women in Kenya aged 15 to 19 and 81 live births per 1000 women aged 15 to 19 in Nairobi (Kenya National Bureau of Statistics et al., 2015).

Analysis of longitudinal data on adolescents living in five cities, including Nairobi, revealed that the transitions to first sex, marriage, and pregnancy are not closely tied; in fact, a higher percent of adolescents living in urban Kenya transition to first pregnancy before first marriage, meaning that premarital births are common (Speizer et al., 2017).

As of year 2022, 14.8 % of girls aged 15–19 in Kenya and 8.4 % of those in Nairobi City have had ever been pregnant, meaning had a live birth, are currently pregnant, and/or reported having had a pregnancy loss (authors' estimations based on the 2022 Kenya DHS). 66.1 % of girls aged 15–19 in Kenya had at least some secondary schooling and 91.0 % were literate, meaning could read a sentence; in Nairobi, 90.1 % of girls aged 15–19 had at least some secondary schooling and 95.7 % were literate (Kenya National Bureau of Statistics and The DHS Program ICF, 2023).

At the national level, 21.0 % of girls aged 15–19 who had not attended secondary school had ever been pregnant, compared to 11.7 % of their peers who had at least some secondary schooling. Further, 32.0 % of girls aged 15–19 who were not literate had ever been pregnant, compared to 13.1 % of their peers who were literate (authors' estimations based on the 2022 Kenya DHS).

### 1.5 Contributions and objectives

This paper uses longitudinal data on adolescent girls in an urban informal settlement in Nairobi, Kenya to explore the following research questions.

1. Are adolescent girls' literacy and numeracy skills, school attendance, grade attainment, and school and non-school friendships independently related to their risk of pregnancy?
2. Is the relationship between adolescent girls' education and risk of pregnancy mediated by their types of friendships?

Based on evidence from other studies, we hypothesize that school attendance, increased grade attainment, and higher levels of literacy and numeracy may be associated with decreased probability of pregnancy among adolescent girls. Furthermore, we posit that any protective effects of girls’ education and learning skills on their risk of pregnancy may be mediated to some extent by their network of friends and may vary depending on whether such friends are male or female, attend school or not, and had been married or given birth.

Understanding the ways in which specific educational factors influence adolescent girls’ risk of pregnancy, and whether and how friendships act independently or as a mediator for this outcome, is important for the purposes of policy and program design. Crucially, it can also highlight where cross-sectoral collaboration can drive better education and health outcomes for girls, young women, and future generations.

## 2 Sample and methods

### 2.1 Data source

This study uses three waves of longitudinal data from the Adolescent Girls Initiative—Kenya (AGI-K) study conducted in Kibera, an urban informal settlement in Nairobi, Kenya between years 2015–2019. AGI-K was a randomized controlled trial that delivered interventions over a two-year period to girls aged 11–15. To assess if and how intervening in early adolescence impacts outcomes for girls, the study tested different packages of a multi-sectoral intervention: Arm 1) Violence Prevention; Arm 2) Violence Prevention + Education; Arm 3) Violence Prevention + Education + Health; and Arm 4) Violence Prevention + Education + Health + Wealth Creation (the full program package). Data were collected at baseline (prior to intervention) in 2015, after the intervention ended in 2017, and in 2019 (two years after the intervention had ended). Further details on the study design and data collection are available in the study protocol (Austrian et al., 2016).

The AGI-K study in Kibera included 2390 girls aged 11–15 at baseline. For the current study, our analytic sample includes girls who were interviewed at all three time points (n = 1993). Differences at baseline between girls in our analytic sample and those lost to follow up are presented in Supplemental Table 1.

### 2.2 Ethics

This study relies on secondary analysis of deidentified data and thus is considered exempt by the Population Council Institutional Review board. The AGI-K study received ethical approval from the Population Council Institutional Review Board, the African Medical and Research Foundation Ethical and Scientific Review Committee, and the Kenyan National Commission for Science, Technology, and Innovation (Austrian et al., 2016).

### 2.3 Measures

#### 2.3.1 Adolescent pregnancy

This study explored experience of pregnancy as the main dependent variable. At round one (2015), respondents were asked if they had ever given birth, were currently pregnant, were ever pregnant when they did not want to be, and/or ever had a miscarriage or stillbirth. At round two (2017) and round three (2019), respondents were asked if they had experienced these events since the last survey round. The birth and pregnancy questions were asked only to those respondents who reported ever having had sex.

We created a binary variable coded as ‘0’ meaning had not experienced a pregnancy since the previous round (by the time of the survey for round one), and ‘1’ meaning had experienced a pregnancy since the previous round (by the time of the survey for round one).

#### 2.3.2 Schooling and learning skills

Four aspects of girls' education were explored as independent variables: numeracy, literacy, highest grade completed, and school attendance. Our explanatory variables capture girls' schooling and learning skills at the exact time of interview. However, experience of pregnancy reported at each survey round would have been in reference to an event that occurred prior to the date of interview. In order to address these temporal concerns, we use lagged independent variables with a lag of one survey round (that is, a lag of a two-year period). This enables us to explore whether reported pregnancy at round two (2017) or round three (2019) depend on girls’ level of numeracy, literacy, school attendance, or grade completion at round one (2015) or round two (2017).

*Numeracy*, specifically girls’ ability to add, subtract, divide and multiply, was assessed at all three rounds using a portion of the Uwezo Kenya National Learning Assessment 2012 tool based on the Kenyan curriculum for Standard 2 (Uwezo, 2011). Round two (2017) included additional more complex problems (worded problems, fractions, percentages) drawn from the Kenya Certificate of Primary Education exam questions. Numeracy scores are based on 20 questions at round one (2015) and 31 questions at round two (2017). For this analysis, numeracy scores at each round are standardized to have a mean of zero and standard deviation of 1 (z scores) to ensure comparability across rounds despite differences in the number of questions administered.

*Literacy* (bilingual literacy) was measured at round one (2015) using a test that assessed the girl's ability to read aloud completely without error two simple sentences in Swahili and two simple sentences in English. Partial reading of a sentence was scored with one point and full reading was scored with three points, for a total of 12 points. The literacy assessment at round two (2017) incorporated reading of short paragraphs (of four sentences each) in addition to the simple sentences. Partial reading of a paragraph was scored with two points and fully reading a paragraph was scored with five points. The highest possible score at round two (2017) is 32. Literacy scores at each round are standardized to have a mean of zero and standard deviation of one.

*Highest grade completed* was self-reported by respondents at each survey round and is treated as a continuous variable.

*School attendance* was self-reported by respondents at each survey round and is coded as a binary variable, ‘0’ meaning did not attend school at any time during the current school year and ‘1’ meaning attended school.

#### 2.3.3 Friendships

As part of a survey module on social networks, girls were asked at each round to report how many good friends they had. The interviewer was given directions to explicitly define a good friend to mean, “someone you can confide in about personal matters and share important information.” Respondents who reported having any good friends were then asked to specify how many of those male or female friends were currently attending school,^1^ had ever been married, and had ever given birth/fathered a child. The interviewer was given directions to first ask all questions specifically about male friends and then ask the same series of questions about female friends.

For this analysis, we examine whether girls have any male friends who were in school; whether girls have any male friends who were not in school; whether girls have any female friends who were in school; and whether girls have any female friends with any of the following characteristics: not in school, ever been married, or ever given birth. All variables are binary and coded as ‘0’ meaning no good friends of this type and ‘1’ meaning at least one good friend of this type.

Since we capture girls' friendships at the exact time of survey, we use lagged independent variables with a lag of one survey round. This enables us to explore whether reported pregnancy at round two (2017) or round three (2019) depends on girls’ friendships at round one (2015) or round two (2017).

#### 2.3.4 Other key covariates

The following covariates were included in the models: g*irls' self-reported age* (a continuous variable), *household wealth score* (a continuous variable),^2^ and whether the respondent had already reported a pregnancy at a previous survey round (a binary variable). Models also adjusted for potential interaction between girls’ age and their assignment to AGI-K intervention arm.

## 3 Methods

We present descriptive statistics on key dependent and independent variables for each study wave.

We follow Baron and Kenny's procedures for mediational hypotheses in order to assess whether the friendship variables mediate the relationship between the education variables and the outcome variable (pregnancy) (Baron & Kenny, 1986). Per Baron and Kenny's procedures, we first explore the association between the education variables and the outcome variable (pregnancy) independent of the potential mediator variables using linear regression models, adjusting for confounders. We also assess the association between the education variables and the potential mediator variables (friendships), adjusting for confounders. To test for partial mediation, we then examine the association between the potential mediator variables (friendships) and the outcome variable (pregnancy). Finally, to test for complete mediation, we determine whether the associations between the education variables and the outcome variable change after including the potential mediators in the model. We estimate the natural direct, natural indirect, and marginal total effects; we use the *Paramed* command in Stata to estimate the proportion of effect mediated (Emsley & Liu, 2013).

Our final model is estimated as follows:

$$Y_{it}=\beta_{0}+\beta_{1}N_{it-1}+\beta_{2}L_{it-1}+\beta_{3}G_{it-1}+\beta_{4}S_{it-1}+{\beta_{5}F_{it-1}+\beta_{6}W_{it-1}+\beta }_{7}X_{it}+\beta_{8}{X_{it}I}_{i}+\beta_{8}P_{it-1}+a_{i}+u_{it}$$

where $Y_{it}$ represents the dependent variable for respondent *i* measured at time *t*; $N_{it-1}$ represents level of numeracy (number of standard deviations below and above the mean) for respondent *i* at time *t*-1; $L_{it-1}$ represents literacy for respondent *i* at time *t*-*1*; $G_{it-1}$ represents level of grade completion for respondent *i* at time *t*-*1*; $S_{it-1}$ represents attendance in school for respondent *i* at time *t*-*1*; $F_{it-1}$ represents friendships for respondent *i* at time *t*-*1*; $W_{it-1}$ represents household wealth score for respondent *i* at time *t*-*1*; $X_{it}$ represents age of respondent *i* at time *t*; ${X_{it}I}_{i}$ represents an interaction between age of respondent *i* at time *t* and respondent's study arm assignment at baseline; $P_{it-1}$ represents whether respondent *i* reported having experienced a pregnancy at time *t-1*; $a_{i}$ is the time invariant individual effect and $u_{it}$ is a random error. Time (t) represents survey wave, where t = 0 is the first survey wave in 2015, t = 1 is the second wave in 2017, and t = 2 is the third wave in 2019.

We estimate the function above using fixed-effects linear probability regression models that remove the time-invariant effects at the individual level. Linear probability regression models were run (rather than logistic) given the relatively low prevalence of the outcome in our sample. We also estimated the function using random-effects regression models. Based on results of Hausman tests comparing estimates from fixed- and random-effects regression models, we reject the null hypothesis that random-effects estimates were consistent; thus, we present only estimates from fixed-effects models. After the final model was fitted, predicted probabilities were calculated using the *margins* command in Stata.

All data were analyzed using Stata version 17.0.

## 4 Results

### 4.1 Descriptive results

Table 1 presents descriptive statistics on respondents’ background characteristics, sexual and reproductive health, education, and friendships, by study round. At baseline, 2.6 % of the sample were aged 9–10; 49.1 % were aged 11–12; 42.7 % were aged 13–14; 5.3 % were aged 15; and 0.3 % were aged 16. The percent of girls who had ever had sex was 1.2 % at round one (2015), 6.2 % by round two (2017), and 17.1 % by round three (2019). Among those who experienced sexual initiation by round two (2017), 44.7 % reported having not used a condom at first sex; among those who experienced sexual initiation by round three (2019), 35.8 % reported having not used a condom at first sex. Experience of pregnancy increased over time: 0.1 % of girls reporting having experienced a pregnancy by round one (2015); 1.7 % of girls reported having experienced a pregnancy between round one (2015) and round two (2017); 5.8 % of girls reported having experienced a pregnancy between round two (2017) and round three (2019). By round three (2019), a total of 6.3 % of girls reported having ever been pregnant. Very few (0.1 %) girls were married by round one (2015) and 1.8 % by round three (2019).

**Table 1:** Descriptive statistics on respondents’ background characteristics and sexual and reproductive health outcomes, by study round.

	Round 1 (2015)	Round 2 (2017)	Round 3 (2019)
**BACKGROUND CHARACTERISTICS**			
**Age of girl at the time of interview**			
*9 to 10 years*	2.6 %	0 %	0 %
*11 to 12 years*	49.1 %	1.9 %	0 %
*13 to 14 years*	42.7 %	43.8 %	1.9 %
*15 years*	5.3 %	25.0 %	15.9 %
*16 to 17 years*	0.3 %	28.8 %	52.8 %
*18 to 20 years*	0 %	0.6 %	29.4 %
**Mean age of girl at the time of interview**	12.6	14.7	16.7
**Marital status**			
*Never married*	99.9 %	99.4 %	96.8 %
*Married or living with partner*	0.05 %	0.3 %	1.8 %
*Separated or divorced*	0.05 %	0.3 %	1.4 %
**Study arm assignment**			
*Arm 1: Violence Prevention Only*	22.3 %	–	–
*Arm 2: Violence + Education*	26.0 %	–	–
*Arm 3: Violence + Education + Health*	26.0 %	–	–
*Arm 4: Violence + Education + Health + Wealth*	25.7 %	–	–
**SEXUAL AND REPRODUCTIVE HEALTH**			
Ever had sex	1.2 %	6.2 %	17.1 %
Did not use a condom at first sex (among those who ever had sex)	(78.3 %)	44.7 %	35.8 %
Ever experienced a pregnancy	0.1 %	1.7 %	6.3 %
Experienced a pregnancy since last interview	0.1 %	1.7 %	5.8 %

Notes: Sample = 1993 respondents. Estimates in parentheses are based on between 25 and 50 cases.

As shown in Table 2, the percent of girls who attended school was high but declined slightly from 98.9 % at round one (2015) to 97.1 % at round two (2017). The mean number of years of schooling completed was 5.7 at round one (2015) and 7.6 at round two (2017). Levels of literacy were also high, with low standard deviation in mean scores at each round. The mean score for numeracy was 18.8 out of 20 questions at round one (2015) and 25.4 out of 31 questions at round two (2017). Standard deviation in scores on the numeracy assessment was low in round one (2015) but higher in round two (2017), indicating that variation in the numeracy scores increased over time.

**Table 2:** Summary statistics on respondents’ education and friendships measured at rounds one and two.

	Round 1 (2015)	Round 2 (2017)
**SCHOOLING AND LEARNING SKILLS**		
Literacy score (mean)	11.7	31.3
Literacy score (standard deviation)	1.5	3.4
Numeracy score (mean)	18.8	25.4
Numeracy score (standard deviation)	2.1	3.9
Attend school in the past year	98.9 %	97.1 %
Highest grade completed	5.7	7.6
**FRIENDSHIPS**		
**Any male friends**	22.3 %	42.9 %
*Any male friends in school*	21.4 %	37.3 %
*Any male friends not in school*	1.8 %	9.2 %
**Any female friends**	95.7 %	95.1 %
*Any female friends in school*	94.4 %	91.6 %
*Any female friends who are not in school, were married, or gave birth*	5.0 %	14.5 %
*Any female friends not in school*	4.0 %	11.4 %
*Any female friends who have been married*	1.1 %	6.8 %
*Any female friends who have given birth*	1.5 %	8.1 %
**Mean number of male friends**	0.7	1.2
*Mean number of male friends in school*	0.6	1.0
*Mean number of male friends not in school*	0.0	0.2
**Mean number of female friends**	3.7	3.3
*Mean number of female friends in school*	3.6	3.1
*Mean number of female friends not in school*	0.1	0.3
*Mean number of female friends who are married*	0.0	0.1
*Mean number of female friends who have given birth*	0.0	0.1

Notes: Sample = 1993 respondents. School attendance and highest grade completed were self-reported by respondents at each survey round. At round one, literacy was measured using a test that assessed the girl's ability to read aloud completely without error two simple sentences in Swahili and two simple sentences in English; partial reading of a sentence was scored with one point and full reading was scored with three points, for a total of 12 points. At round two, the literacy assessment incorporated reading of short paragraphs (of four sentences each) in addition to the simple sentences; partial reading of a paragraph was scored with two points and fully reading a paragraph was scored with five points, with 32 being the highest possible score. Numeracy score was derived based on responses to a total of 20 questions at round 1 and 31 questions at round 2. Descriptive statistics on girls' schooling, learning skills, and friendships at round three are not shown because our analysis uses lagged independent variables with data only from rounds one and two.

The proportion of girls who reported having any male friends increased from 22.3 % in round one (2015) to 42.9 % in round two (2017). Among the girls who reported having any male friends, the majority referred to male friends who were currently attending school. At round one (2015), only 1.8 % reported having any male friends who were not enrolled in school; at round two (2017), 9.2 % reported out-of-school male friends.

While the percent of girls who reported having any good male friends increased over time, the percent who reported having any good female friends was consistent at both rounds. However, the types of female friendships appear to have changed as girls aged. The percent of girls who reported having female friends who were not in school, had ever been married or had ever given birth increased from 5.0 % at round one (2015) to 14.5 % at round two (2017).

As shown in Table 3, we find evidence of significant associations between girls’ schooling and learning skills (the independent variables) and their friendships (the potential mediator variables) at round one (2015) and at round two (2017). At both rounds, girls who reported having any male friends who were not enrolled in school were significantly less likely to attend school themselves. Girls who reported having any female friends who were not enrolled in school, had been married, or given birth were also significantly less likely to attend school at both rounds. At round two (2017), girls who had any female friends in school or who had any male friends in school were significantly more likely to have higher numeracy and literacy scores relative to the average.

**Table 3:** Descriptive statistics on girls’ schooling and learning skills in relation to their friendships at round one (2015) and round two (2017).

	Attended school				Highest grade completed				Numeracy z score			Literacy z score		
	(%)				(mean)				(mean)			(mean)		
	Round 1		Round 2		Round 1		Round 2		Round 1	Round 2		Round 1	Round 2	
**Male friends in school**														
None	98.9		96.7		5.60	***	7.30	***	−0.01	−0.04	**	0.02	−0.02	**
Any	99.1		97.8		6.00		7.90		0.05	0.08		0.04	0.09	
**Male friends not in school**														
None	99.0	**	97.9	***	5.70	***	7.50	***	0.00	0.00		0.02	0.02	
Any	(94.4)		89.9		(6.80)		8.40		(0.07)	0.02		(0.16)	0.00	
**Female friends in school**														
None	95.5	***	87.4	***	5.90	*	7.40	*	0.07	−0.37	***	−0.05	−0.29	***
Any	99.2		98.0		5.60		7.60		0.00	0.04		0.03	0.04	
**Female friends out of school, married or given birth**														
None	99.3	***	98.0	***	5.70	**	7.50	***	0.01	0.00		0.03	0.02	
Any	91.9		91.6		6.10		8.00		−0.12	0.02		0.00	−0.04	

Notes: School attendance and highest grade completed were self-reported by respondents at each survey round. Literacy and numeracy scores are standardized to have a mean of zero and standard deviation of 1 (z scores) to ensure comparability across rounds despite differences in the number of questions administered. At round one, literacy was measured using a test that assessed the girl's ability to read aloud completely without error two simple sentences in Swahili and two simple sentences in English; partial reading of a sentence was scored with one point and full reading was scored with three points, for a total of 12 points. At round two, the literacy assessment incorporated reading of short paragraphs (of four sentences each) in addition to the simple sentences; partial reading of a paragraph was scored with two points and fully reading a paragraph was scored with five points, with 32 being the highest possible score. Numeracy score was derived based on responses to a total of 20 questions at round 1 and 31 questions at round 2. Descriptive statistics on girls' schooling and learning skills in relation to their friendships at round three are not shown because our analysis uses lagged independent variables with data only from rounds one and two. Sample = 1993 respondents. Estimates in parentheses are based on between 25 and 50 cases. p-values for differences between groups in the percent of girls who attended school are from chi-square tests; p-values for differences in means between groups are from t-tests. ***p < 0.001; **p < 0.01; *p < 0.05.

### 4.2 Pregnancy

Table 4 presents the coefficients obtained from estimating the equation for pregnancy using individual-level fixed-effects regression models. As shown in Model I, after adjusting for key background covariates, attending school decreases the probability of pregnancy by nine percentage points; completing an additional year of schooling decreases the probability of pregnancy by three percentage points; and a one-point standard deviation increase in numeracy score decreases the probability of pregnancy by one percentage point.

**Table 4:** Results of fixed-effects linear probability regression models. Effects of girls' schooling, learning skills, and friendships on the probability of experiencing a pregnancy between rounds.

	(I) Including schooling and learning variables	(II) Including friendship variables	(III) Including schooling, learning, and friendship variables
	Coef.	Coef.	Coef.
**Literacy z-score**	−0.01		−0.01
**Numeracy z score**	−0.01*		−0.01*
**Highest grade completed**	−0.03**		−0.03**
**Attended school in the past year** (Ref. group = Did not attend school)	−0.09**		−0.09**
**Had any male friends in school** (Ref. group = None)		0.01	0.01
**Had any male friends not in school** (Ref. group = None)		0.05**	0.04*
**Had any female friends in school** (Ref. group = None)		0.01	0.01
**Had any female friends out of school, married or given birth** (Ref. group = None)		0.01	0.01
**Girl's age**	0.06***	0.03***	0.06***
**Girl's age × Study arm assignment**			
Violence Prevention Only	−0.01	−0.01	−0.01
Violence + Education	0.0	0.0	0.0
Violence + Education + Health	−0.01	−0.01	−0.01
**Household wealth score**	0.0	0.0	0.0
**Reported having experienced a pregnancy at prior round** (Ref. group = No prior pregnancy)	−0.44***	−0.36***	−0.45***

Notes: Literacy and numeracy scores are standardized to have a mean of zero and standard deviation of 1 (z scores) to ensure comparability across rounds despite differences in the number of questions administered. Sample = 1993 respondents (3887 observations). Coef. = coefficients. ***p < 0.001; **p < 0.01; *p < 0.05.

Table 4 also presents coefficients from models estimating the probability of experiencing a pregnancy based on types of friendships, adjusted first for key background characteristics (Model II) and then also for schooling and learning factors (Model III). As shown in Model II, having any male friends who do not attend school increases girls' probability of experiencing a pregnancy by five percentage points. We find no evidence that other friendships—specifically friendships with males who attend school, with females who attend school, or with females who do not attend school, have been married, or have given birth—increase or decrease girls’ probability of experiencing a pregnancy.

The strong protective effects of girls' numeracy, grade completion, and school attendance remained unchanged even after adjusting for friendship factors (Model III). The association between pregnancy and male friends outside of school remained but was slightly weakened after adjusting for girls' education: having any male friends who do not attend school increases girls’ probability of experiencing a pregnancy by four percentage points (Model III).

To illustrate the risk of pregnancy that girls face, we estimated predicted probabilities based on Model III. Holding other covariates constant at their mean value, the predicted probability of experiencing a pregnancy within the next two years is 12.9 % for girls who do not attend school compared to 3.5 % for girls who attend school. For in-school girls, the predicted probability of experiencing a pregnancy within the next two years is higher (4.6 % for girls whose numeracy score is one standard deviation below the mean than for girls whose numeracy score is one standard deviation above the mean (2.4 %). The predicted probability of experiencing a pregnancy within the next two years for girls who have any out-of-school male friends is 7.5 % for in-school girls and 16.9 % for out-of-school girls.

Finally, while numeracy, grade completion, and school attendance had significant direct effects on pregnancy, we find no evidence that the effects of these independent variables (education) on the outcome (pregnancy) were mediated by the friendship factors. Additional mediation analyses that did not account for fixed effects produced similar results (Supplemental Table 2).

### 4.3 Robustness considerations

We noted correlation in girls' responses to questions about whether they have any friends who were not in school, who had ever been married, or who had ever given birth (Supplemental Table 3). Due to the design of the three questions, there can also be overlap in girls’ responses, meaning they refer to the same friend. For the purpose of our analyses, we combined responses into one variable capturing whether girls have any female friends who were either not in school, ever married, or had given birth. However, we also explored these friendships as three separate covariates; the overall direction of effect remains the same and their associations with the outcomes are statistically insignificant (Supplemental Table 4).

Given the large effect of school attendance and that only a small percentage of girls were out of school, we also estimated the models on the subsample of girls who were in school in the previous round. We find that the coefficients for numeracy and grade attainment remain similar in size and significant but the coefficient for having any out-of-school male friends is smaller and statistically insignificant (Supplemental Table 5).

## 5 Discussion

Overall, in this context, adolescent girls' schooling, learning skills, and friendships with out-of-school males independently influence their risk of pregnancy – with education being protective and out-of-school males, a potential risk. The longitudinal nature of the data and the results of fixed effects regression analyses suggest that these relationships are causal. In terms of our initial hypothesis, we find that the protective effects of girls’ school attendance, grade completion and numeracy skills on their risk of pregnancy are not mediated by their friendships. However, we also observe that out-of-school girls are disproportionately exposed to out-of-school males and, therefore, to early pregnancy.

In all models, higher levels of literacy were associated with lower probability of pregnancy. However, these associations were statistically insignificant, in part because the literacy assessments used were not challenging enough to pick up variation among the sample (Table 2). We find that attending school, completing additional years of schooling, and having higher numeracy skills relative to one's peers significantly decreases the probability of experiencing a pregnancy. The protective effects of numeracy skills and grade attainment remain even after controlling for girls' schooling status (in-school vs. out-of-school), suggesting that the benefits of education are an asset for girls even after leaving school.

Our findings are consistent with the results of other studies that demonstrate the protective effects of education and school performance on girls' risk of pregnancy (Bhuwania et al., 2023; Marteleto et al., 2008). Clark and Mathur's study on adolescents in urban Kenya found that girls who were enrolled in school and academically on-track were the least likely to be sexually active whereas girls who had dropped out of school faced the highest risk of sexual initiation (Clark & Mathur, 2012); we observe a similar pattern when examining pregnancy as an outcome.

Having any male friends who do not attend school increases the probability of pregnancy among girls even after adjusting for schooling and learning skills as well as all other types of friendships. However, close friendships with out of school males are more frequently reported by out-of-school girls than in-school girls, suggesting the risk these friendships pose is predominantly experienced by girls who have left school. For in-school girls, the pregnancy risk associated with out-of-school males is lower, and not significant. Unfortunately, we do not have data on the characteristics of out-of-school males and thus do not know whether they were typically older.

While other studies have shed light on the important influence of peer groups relative to schooling (Barker et al., 2019), we found that other types of friendships, including those with males who attend school, were not associated with experiencing a pregnancy in this context. It appears that peer group norms or behaviors did not increase or decrease risk. Importantly, our results indicate that the strong protective effects of girls’ numeracy skills, grade completion, and school attendance on risk of pregnancy do not appear to have been mediated by their types of friendships.

We acknowledge several important limitations of our study. First, data for this study were collected through a randomized controlled trail that involved the implementation of a two-year intervention that was shown to have impact on girls' sexual debut, pregnancy, primary school completion, and transition to secondary school (Kangwana et al., 2022). While our analysis controlled for an interaction between age and girls' assignment to the different study arms, our findings are based on a sample of girls subject to an intervention and are thus not generalizable beyond this setting. Second, this analysis relies on self-reported data, which can be affected by recall or social desirability bias. This is of particular concern regarding sensitive behaviors such as sex and early pregnancy (Kelly et al., 2013; Mensch et al., 2003). Third, our literacy assessment found fairly high levels of literacy and little variation; our numeracy assessment also found little variation at round one (2015). If this is due to an inadequate assessment, rather than actual skills, we may have failed to capture the impact of literacy or underestimated the magnitude of the effect of numeracy on early pregnancy. Finally, our analysis may be limited by the lack of available data on other potentially important confounders, including on the nature of the girl's relationship with a male friend.

Despite these limitations, this analysis of longitudinal data collected over the pivotal period from early to late adolescence provides useful, nuanced insight on the ways in which girls' education and social networks operate independently to influence girls' risk of early pregnancy. It takes a substantial step beyond cross-sectional analyses by accounting for all time-constant individual effects and lagging covariates and thus addressing reverse causality concerns. We find significant protective effects of school attendance, grade attainment, and numeracy skills on girls’ pregnancy; we also find that having any close friendships with out-of-school males increases the probability of experiencing a pregnancy. These effects are independent and temporally ordered, suggesting causality.

## 6 Conclusion

Our findings suggest that keeping girls in school and ensuring that they learn are not only important outcomes in and of themselves, but they have direct sexual and reproductive health benefits. At the same time, numeracy and grade attainment do not remove sexual and reproductive health risks that out-of-school male friendships bring, especially for out-of-school girls. Thus, while quality schooling that ensures girls remain in school and develop literacy and numeracy skills helps to reduce their risk of pregnancy, education did not eliminate the independent risk that operates through friendships with out-of-school males. These risks may need to be addressed through other interventions that address vulnerabilities and disparities in other dimensions of girls’ lives.

A better understanding of the circumstances under which girls became pregnant in this context—specifically, whether these pregnancies were intended or unintended, and whether they occurred in the context of wanted or unwanted sex—could help inform the types of interventions needed. In settings with widespread school enrollment like that of the AGI-K study in Kibera, qualitative research might shed light on the dynamics of out-of-school girls' relationships with out-of-school males and therefore help to inform choice of intervention (i.e., interventions that address power inequalities in relationships and sexual and reproductive health, shift inequitable gender norms, eliminate child marriage, or reduce girls’ economic vulnerability). For girls who are enrolled in school, keeping them in school and ensuring they learn are critical goals in their own right, but they also may contribute substantially to preventing pregnancy; more research is needed on what interventions are most effective in ensuring girls transition from primary to secondary school.

Future research can build on our findings by exploring other potential mediators of the effects of education—specifically the causal effects of learning skills, school attendance, and grade attainment—on risk of pregnancy and other sexual and reproductive health outcomes. By untangling the pathways between education and sexual and reproductive health, we can illuminate how education protects the health and well-being of young people, especially girls; understand the intersections with other multi-sectoral determinants; and better define the implications for policies and programs.

## Funding source

This work was supported by a grant from the 10.13039/100000865Bill & Melinda Gates Foundation (Investment ID INV-005386).

## Declaration of generative AI in scientific writing

None to declare.

## Submission declaration and verification

This manuscript has not been published previously and is not currently under consideration for publication elsewhere.

## CRediT authorship contribution statement

**Christina Misunas:** Conceptualization, Formal analysis, Methodology, Writing – original draft, Writing – review & editing. **Erica Soler-Hampejsek:** Conceptualization, Methodology, Writing – review & editing. **Beth Kangwana:** Writing – review & editing. **Nicole A. Haberland:** Conceptualization, Methodology, Project administration, Resources, Supervision, Writing – review & editing.

## Declaration of competing interest

None to declare.

## Data Availability

Data are available for download through Harvard Dataverse.

## Footnotes

1 For each respondent, we were able to subtract the reported number of male/female friends currently in school from the total reported number of male/female friends to determine the number of male/female friends not in school at the time of survey.

2 Household wealth score was derived based on responses at each survey round from a household survey answered by the household head or other adult household member to questions about possession of household items such as television, watch, mosquito net; ownership of livestock and agricultural land; the number of sleeping rooms; and whether the family experienced days without food in the past month, and had enough savings in case they needed. Responses to each household item/condition were dichotomized and principal components analysis was used to calculate household wealth scores for each adolescent girl respondent.
