# Supplementary material for: Do adolescent girls’ education and friendships have independent effects on early pregnancy? Results of a mediation analysis from a longitudinal cohort study in Nairobi, Kenya
Source: SSM Popul Health. 2024 Feb 1;25:101618. doi: 10.1016/j.ssmph.2024.101618 (PMC10901828; doi:10.1016/j.ssmph.2024.101618)
Supplement: Multimedia component 1 [file mmc1.docx]

| **Supplemental - Table 1:** Differences in background characteristics, sexual and reproductive health, education, and friendships at baseline between girls interviewed at all three rounds (analytic sample) and those lost to follow up. | | |
| --- | --- | --- |
|  | **Interviewed at all three rounds** | **Interviewed at Round One and lost to follow up** |
| **BACKGROUND CHARACTERISTICS** |  |  |
| **Age of girl*** |  |  |
| *9 to 10 years* | 2.6 % | 3.3 % |
| *11 to 12 years* | 49.1 % | 41.1 % |
| *13 to 14 years* | 42.7 % | 49.9 % |
| *15 years* | 5.3 % | 5.3 % |
| *16 to 17 years* | 0.3 % | 0.5 % |
| **Mother’s level of education** |  |  |
| *None or some primary* | 29.8 % | 32.3 % |
| *Completed primary or some secondary* | 34.9 % | 32.1 % |
| *Secondary or higher* | 33.6 % | 33.3 % |
| *Don’t know* | 1.7 % | 2.3 % |
| **Father’s level of education*** |  |  |
| *None or some primary* | 15.7 % | 16.1 % |
| *Completed primary or some secondary* | 17.4 % | 24.8 % |
| *Secondary or higher* | 61.6 % | 54.4 % |
| *Don’t know* | 5.3 % | 4.7 % |
| **Marital status** |  |  |
| *Never married* | 99.9 % | 99.5 % |
| *Married or living with partner* | 0.1 % | 0.3 % |
| *Separated or divorced* | 0.1 % | 0.3 % |
| **Study arm assignment***** |  |  |
| *Arm 1: Violence Prevention Only* | 22.3 % | 38.5 % |
| *Arm 2: Violence + Education* | 26.0 % | 18.6 % |
| *Arm 3: Violence + Education + Health* | 26.0 % | 22.9 % |
| *Arm 4: Violence + Education + Health + Wealth* | 25.7 % | 19.9 % |
| **SEXUAL AND REPRODUCTIVE HEALTH** |  |  |
| Ever had sex** | 1.2 % | 2.8 % |
| Ever experienced a pregnancy | 0.1 % | 0.5 % |
| **SCHOOLING AND LEARNING SKILLS** |  |  |
| Literacy score (mean) | 11.7 | 11.4 |
| Numeracy score (mean) | 18.8 | 18.7 |
| Attend school in the past year | 98.9 % | 99.2 % |
| Highest grade completed | 5.7 | 5.7 |
| **FRIENDSHIPS** |  |  |
| *Any male friends in school* | 21.4 % | 22.2 % |
| *Any male friends not in school*** | 1.8 % | 4.1 % |
| *Any female friends in school* | 94.4 % | 92.7 % |
| *Any female friends who are not in school, were married, or gave birth** | 5.0 % | 7.6 % |
| *Any female friends not in school* | 4.0 % | 6.1 % |
| *Any female friends who have been married* | 1.1 % | 1.5 % |
| *Any female friends who have given birth** | 1.5 % | 3.0 % |
| Observations | 1,993 | 397 |
| Notes: p-values for differences in proportions between groups are from chi-square tests; p-values for differences in means between groups are from t-tests.  *** p < 0.001; ** p < 0.01; * p < 0.05 | | |

| **Supplemental - Table 2:** Results of mediation analysis. The effects of numeracy, school attendance, and grade completion on probability of experiencing a pregnancy between rounds, adjusting for potential mediation by friendships. | | | | | | | |
| --- | --- | --- | --- | --- | --- | --- | --- |
| **Potential Mediator** | **Effect of numeracy on probability of experiencing a pregnancy** | Coef. | **Effect of school attendance on probability of experiencing a pregnancy** | | Coef. | **Effect of highest grade completed on probability of experiencing a pregnancy** | Coef. |
| *Had any male friends in school* | NDE | -0.011*** | NDE | -0.319*** | | NDE | -0.004 |
|  | NIE | 0.000 | NIE | 0.002* | | NIE | 0.001** |
|  | MTE | -0.011*** | MTE | -0.317*** | | MTE | -0.003 |
| *Had any male friends out of school* | NDE | -0.011*** | NDE | -0.319*** | | NDE | -0.004 |
|  | NIE | 0.000 | NIE | -0.010*** | | NIE | 0.001*** |
|  | MTE | -0.011*** | MTE | -0.330*** | | MTE | -0.002 |
| *Had any female friends in school* | NDE | -0.011*** | NDE | -0.319*** | | NDE | -0.004 |
|  | NIE | 0.000 | NIE | -0.005** | | NIE | 0.000 |
|  | MTE | -0.011*** | MTE | -0.324*** | | MTE | -0.004 |
| *Had any female friends out of school, married or given birth* | NDE | -0.011*** | NDE | -0.319*** | | NDE | -0.004 |
|  | NIE | 0.000 | NIE | -0.004* | | NIE | 0.000 |
|  | MTE | -0.011*** | MTE | -0.323*** | | MTE | -0.003 |
| Notes: NDE refers to natural direct effect; NIE refers to natural indirect effects; MTE refers to the marginal total effect. Coef. = coefficients. Analysis was performed using the paramed command in Stata. It does not account for fixed effects or potential interaction between age of girl and study arm assignment.  *** p < 0.001; ** p < 0.01; * p < 0.05 | | | | | | | |

| **Supplemental - Table 3:**  Tetrachoric correlations for dichotomous friendship variables based on girls’ responses at round one (2015) and round two (2017). | | | | | | |
| --- | --- | --- | --- | --- | --- | --- |
|  | **Friendships at round one (2015)** | | | | | |
|  | Had any male friends in school | Had any male friends not in school | Had any female friends in school | Had any female friends not in school | Had any female friends who were married | Had any female friends who had given birth |
| Had any male friends in school | 1.00 |  |  |  |  |  |
| Had any male friends not in school | 0.29 | 1.00 |  |  |  |  |
| Had any female friends in school | 0.18 | -0.24 | 1.00 |  |  |  |
| Had any female friends not in school | 0.07 | 0.43 | -0.55 | 1.00 |  |  |
| Had any female friends who were married | 0.18 | 0.48 | -0.27 | 0.62 | 1.00 |  |
| Had any female friends who had given birth | 0.02 | 0.33 | -0.29 | 0.67 | 0.91 | 1.00 |
|  | **Friendships at round two (2017)** | | | | | |
|  | Had any male friends in school | Had any male friends not in school | Had any female friends in school | Had any female friends not in school | Had any female friends who were married | Had any female friends who had given birth |
| Had any male friends in school | 1.00 |  |  |  |  |  |
| Had any male friends not in school | 0.10 | 1.00 |  |  |  |  |
| Had any female friends in school | 0.46 | 0.04 | 1.00 |  |  |  |
| Had any female friends not in school | 0.06 | 0.54 | -0.28 | 1.00 |  |  |
| Had any female friends who were married | 0.03 | 0.38 | -0.19 | 0.89 | 1.00 |  |
| Had any female friends who had given birth | 0.11 | 0.38 | -0.15 | 0.83 | 0.95 | 1.00 |
| Note: Sample = 1,993 respondents. | | | | | | |

| **Supplemental - Table 4**: Results of fixed-effects linear probability regression models. Effects of girls' schooling, learning skills, and friendships on the probability of experiencing a pregnancy between rounds. | | | | | | | | |
| --- | --- | --- | --- | --- | --- | --- | --- | --- |
|  | (II) Including friendship variables | | | | (III) Including schooling, learning, and friendship variables | | | |
|  | Coef. |  | |  | Coef. |  | |  |
| **Literacy z-score** |  | |  |  | -0.01 | |  |  |
| **Numeracy z score** |  | |  |  | -0.01* | |  |  |
| **Highest grade completed** |  | |  |  | -0.03** | |  |  |
| **Attended school in the past year** (Ref. group=Did not attend school) |  | |  |  | -0.10** | |  |  |
| **Had any male friends in school** (Ref. group=None) | 0.01 | |  |  | 0.01 | |  |  |
| **Had any male friends not in school** (Ref. group=None) | 0.05** | |  |  | 0.04* | |  |  |
| **Had any female friends in school** (Ref. group=None) | 0.01 | |  |  | 0.01 | |  |  |
| **Had any female friends not in school** (Ref. group=None) | 0.00 | |  |  | 0.00 | |  |  |
| **Had any female friends who were married** (Ref. group=None) | 0.01 | |  |  | 0.02 | |  |  |
| **Had any female friends who had given birth** (Ref. group=None) | 0.01 | |  |  | 0.01 | |  |  |
| **Girl’s age** | 0.03*** | |  |  | 0.06*** | |  |  |
| **Girl’s age * Study arm assignment** |  | |  |  |  | |  |  |
| Violence Prevention Only | -0.01 | |  |  | -0.01 | |  |  |
| Violence + Education | 0.00 | |  |  | 0.00 | |  |  |
| Violence + Education + Health | -0.01 | |  |  | -0.01 | |  |  |
| **Household wealth score** | 0.00 | |  |  | 0.00 | |  |  |
| **Reported having experienced a pregnancy at prior round** (Ref. group=No prior pregnancy) | -0.37*** | |  |  | -0.45*** | |  |  |
| Notes: Literacy and numeracy scores are standardized to have a mean of zero and standard deviation of 1 (z scores) to ensure comparability across rounds despite differences in the number of questions administered. Sample = 1,993 respondents (3,887 observations). Coef. = coefficients. *** p < 0.001; ** p < 0.01; * p < 0.05 | | | | | | | | |

| **Supplemental - Table 5**: Results of fixed-effects linear probability regression models. Among girls who were in-school, the effects of girls' grade attainment, learning skills, and friendships on the probability of experiencing a pregnancy between rounds. | | | |
| --- | --- | --- | --- |
|  | (I) Including learning variables | (II) Including friendship variables | (III) Including learning, and friendship variables |
|  | Coef. | Coef. | Coef. |
| **Literacy z-score** | -0.01 |  | -0.01 |
| **Numeracy z score** | -0.01** |  | -0.01** |
| **Highest grade completed** | -0.03* |  | -0.03* |
| **Had any male friends in school** (Ref. group=None) |  | 0.01 | 0.01 |
| **Had any male friends not in school** (Ref. group=None) |  | 0.02 | 0.02 |
| **Had any female friends in school** (Ref. group=None) |  | 0.00 | 0.00 |
| **Had any female friends out of school, married or given birth** (Ref. group=None) |  | 0.01 | 0.01 |
| **Girl’s age** | 0.05*** | 0.02*** | 0.05*** |
| **Girl’s age * Study arm assignment** |  |  |  |
| Violence Prevention Only | -0.01 | -0.01 | -0.01 |
| Violence + Education | 0.00 | 0.00 | 0.00 |
| Violence + Education + Health | 0.00 | -0.01 | 0.00 |
| **Household wealth score** | 0.00 | 0.00 | 0.00 |
| **Reported having experienced a pregnancy at prior round** (Ref. group=No prior pregnancy) | -0.30*** | -0.31*** | -0.32*** |
| Notes: Literacy and numeracy scores are standardized to have a mean of zero and standard deviation of 1 (z scores) to ensure comparability across rounds despite differences in the number of questions administered. Sample = 1,976 respondents (3,813 observations). Coef. = coefficients. *** p < 0.001; ** p < 0.01; * p < 0.05 | | | |
